# High-throughput inclined scanning three-dimensional X-ray diffraction microscopy *via*dual line–beam X-point scanning

**DOI:** 10.1107/S2052252526004033

**Published:** 2026-05-18

**Authors:** Jaemyung Kim, Yujiro Hayashi, Makina Yabashi

**Affiliations:** a RIKEN SPring-8 Center, 1-1-1 Kouto, Sayo-cho, Sayo-gun, Hyogo, 679-5148, Japan; Harima Institute, Japan

**Keywords:** dual line–beam X-point scanning, *i*-S3DXRD, orientation mapping, 3DXRD

## Abstract

Recently developed inclined scanning three-dimensional X-ray diffraction (*i*-S3DXRD) microscopy enables the non-destructive mapping of crystal orientations in planar-shaped specimens. However, its inherently slow raster-scanning procedure limits its practical applicability. In this work, we achieve a 21-fold reduction in the number of scans, from 51 × 37 to 51 + 37, by introducing a dual line–beam X-point (DLX) scanning mode in *i*-S3DXRD. Here, 51 and 37 correspond to the numbers of scan steps along the *z* and *x* directions under horizontally and vertically elongated X-ray beam illumination, respectively. The DLX approach selectively enhances diffraction peaks corresponding to the inter­section point (X-point) through pixel-wise multiplication of two diffraction images acquired under horizontally and vertically elongated incident beams. Using the reorganized data, we performed multigrain indexing and found that the resulting orientation map agrees with that ob­tained from conventional point–beam raster scanning, demonstrating the validity and time efficiency of the DLX scanning mode.

## Abstract 2

We present a dual line–beam X-point scanning method for inclined scanning three-dimensional X-ray diffraction microscopy that significantly reduces measurement time while providing three-dimensional orientation maps com­parable to those ob­tained by conventional point–beam raster scanning.

## 1. Introduction

Scanning three-dimensional X-ray diffraction (S3DXRD) microscopy enables the non-destructive characterization of the microstructure of polycrystalline materials (Hayashi *et al.*, 2015 ▸; Hayashi *et al.*, 2019 ▸; Henningsson *et al.*, 2020 ▸; Henningsson *et al.*, 2024 ▸; Hektor *et al.*, 2019 ▸; Shukla *et al.*, 2024*a* ▸; Shukla *et al.*, 2024*b* ▸). Unlike conventional three-dimensional X-ray diffraction (3DXRD) (Juul *et al.*, 2017 ▸; Juul *et al.*, 2020 ▸; Margulies *et al.*, 2001 ▸; Oddershede *et al.*, 2010 ▸; Poulsen *et al.*, 2001*a* ▸; Poulsen *et al.*, 2003 ▸; Poulsen & Fu, 2003 ▸; Poulsen *et al.*, 2012 ▸; Schmidt, 2014 ▸; Winther *et al.*, 2017 ▸) or high-energy diffraction microscopy (HEDM) (Bernier *et al.*, 2011 ▸; Li *et al.*, 2012 ▸; Lienert *et al.*, 2011 ▸; Pokharel *et al.*, 2014 ▸), S3DXRD employs a focused point X-ray beam for diffraction image acquisition. Conical slits reduce the gauge volume, blocking diffraction from outside the target region and minimizing peak overlap, thereby allowing the ob­servation of thick specimens (Hayashi *et al.*, 2019 ▸).

During raster scanning, the specimen rotates continuously, and diffraction signals are integrated over constant angular inter­vals. After scanning, the orientations of individual voxels – each corresponding to an independent crystallite – are reconstructed through multigrain indexing. Grains are then defined by grouping connected voxels within a misorientation threshold, which is the procedure in non-tomographic S3DXRD (Kim *et al.*, 2026*a* ▸). In tomographic S3DXRD, grain morphology is ob­tained through filtered back-projection (FBP) of a sinogram constructed from diffraction peak intensities across the raster scan (Henningsson *et al.*, 2020 ▸; Henningsson *et al.*, 2024 ▸; Hektor *et al.*, 2019 ▸; Shukla *et al.*, 2024*a* ▸; Shukla *et al.*, 2024*b* ▸; Zhang *et al.*, 2025 ▸).

Although both non-tomographic and tomographic S3DXRD provide high-resolution orientation maps, they are not well suited for high-aspect-ratio products (Kim *et al.*, 2026*b* ▸), such as printed circuit boards (PCBs) or metal plates. In these cases, vertical rotation leads to strong X-ray absorption at certain angles, resulting in incom­plete G-vector sets for non-tomographic S3DXRD or incom­plete sinograms for tomographic S3DXRD. Consequently, the reconstruction exhibits a low com­pleteness factor in the non-tomographic case or distorted grains in the tomographic case.

This geometrical limitation also affects parallel X-ray com­puted tomography (CT) (Cnudde & Boone, 2013 ▸; Kalender, 2006 ▸), making it suitable only for cylindrical specimens. However, this restriction can be overcome using inclined rotation geometries, such as com­puterized lamino­graphy (CL) (Gondrom *et al.*, 1999 ▸; Hoshino *et al.*, 2011 ▸; Moore *et al.*, 2002 ▸), in which the sample rotation axis is inclined relative to the X-ray beam. The inclined geometry mitigates excessive X-ray absorption and enables tomography of plate-like specimens *via* FBP over the full rotation angles. Consequently, lamino­graphic rotation can accommodate both cylindrical pillar-shaped samples and plate-like specimens, providing a key advantage over vertical rotation geometry.

Similarly, inclined scanning three-dimensional X-ray dif­frac­tion (*i*-S3DXRD) microscopy – a recently developed vari­ant of S3DXRD – enables the non-destructive characterization of plate-like specimens (Kim *et al.*, 2026*b* ▸). In *i*-S3DXRD, the lamino­graphic rotation geometry inherently avoids massive X-ray absorption, allowing diffraction image acquisition across the full angular range. According to the report, the orientation maps ob­tained by *i*-S3DXRD agree well with electron backscatter diffraction (EBSD) measurements. However, the acquisition time remains long (∼12 h), which is impractical for general applications. The situation becomes even more challenging for specimens with weak diffraction signals, which require longer exposure. Therefore, a faster approach that can achieve reconstruction quality com­parable to *i*-S3DXRD is needed.

In this work, we demonstrate a significant reduction in scanning time by implementing the dual line–beam X-point (DLX) scanning mode within the *i*-S3DXRD framework. In this approach, diffraction images ob­tained under horizontally and vertically extended X-ray beam illumination are multiplied, enhancing the diffraction signals at the X-point. The multigrain indexing results ob­tained from the reorganized dataset are consistent with those reconstructed from conventional raster scans, validating the DLX scanning mode. This method provides a practical way toward the high-throughput non-destructive characterization of plate-like polycrystalline materials, while preserving the accuracy com­parable to *i*-S3DXRD.

## 2. Method

### 2.1. Principle

**Figure 1:**
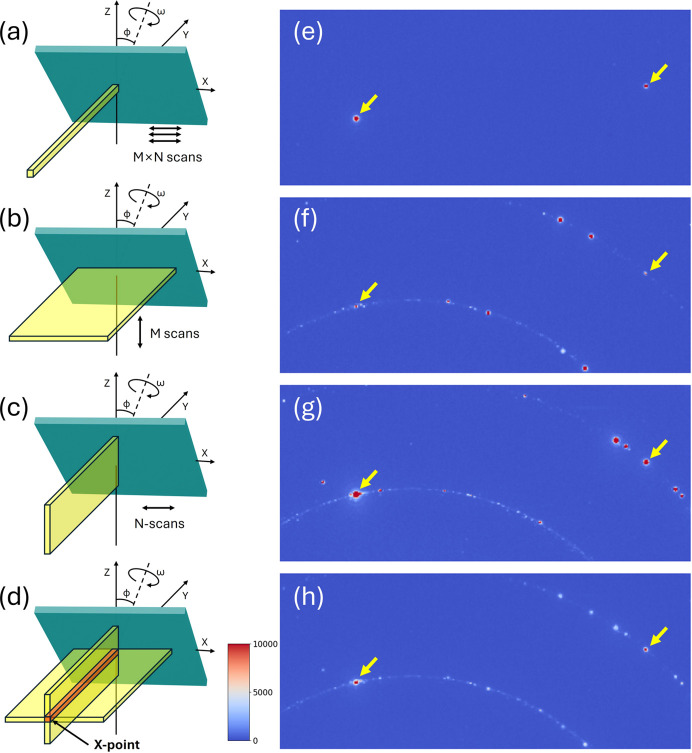
Illustration of conventional raster scanning and DLX scanning. (*a*) Conventional *i*-S3DXRD raster scanning with a point beam, in which the specimen is translated in the *z* and *x* directions, requiring *M* × *N* acquisitions. DLX scanning using a horizontally elongated beam (*b*) and a vertically elongated beam (*c*) covers the entire specimen volume with *M* + *N* acquisitions. (*d*) Multiplication of the two images selectively enhances the diffraction signal at the X-point. X-ray diffraction patterns of the α-Fe steel plate ob­tained with the conventional point beam (*e*), the horizontally elongated beam (*f*) and the vertically elongated beam (*g*), together with their pixel-wise multiplication (*h*), show that diffraction peaks at the X-points become more pronounced after multiplication of the two images, as indicated by the yellow arrows.

An illustration of raster scanning with a point beam is shown in Fig. 1 ▸(*a*). The point beam illuminates the specimen along the *y* direction, and grains satisfying Bragg’s condition produce diffraction signals on the detector. To cover the full field of view with a point beam, the (*X*, *Z*) stage must be translated step-by-step. If the stage translates *M* times along *z* and *N* times along *x*, with an acquisition time *t* at each position, the total measurement time becomes *M* × *N* × *t*. This time increases rapidly with larger *M* and *N*, making raster scanning impractical for wide fields of view.

For example, it took approximately 4.5 h to cover a region of ±100 µm in both the *x* and *z* directions with a step size of 10 µm using *i*-S3DXRD (Kim *et al.*, 2023*a* ▸). Expanding the scan range to ±200 µm for a wider field of view increases the total measurement time to approximately 18 h. This corresponds to a measurement throughput of roughly one sample per day. Given the limited availability of synchrotron beamtime, such a rate is not practical for systematic studies over wide fields of view or multiple samples. This limitation highlights the need for faster scanning approaches that can provide com­parable three-dimensional orientation maps within a sig­nificantly reduced measurement time.

Figs. 1 ▸(*b*)–(*d*) illustrate the principle of the proposed DLX scanning method. When a horizontally elongated X-ray beam illuminates the specimen, as shown in Fig. 1 ▸(*b*), *M* translation steps along *z* are required to cover the field of view. Conversely, illumination with a vertically elongated beam, shown in Fig. 1 ▸(*c*), requires *N* translation steps along *x*. These two illumination modes irradiate the same specimen position twice [Fig. 1 ▸(*d*)], which we refer to as the X-point. Therefore, pixel-wise multiplication of the two images acquired under horizontally and vertically elongated beam illumination selectively enhances the diffraction signal at the X-point, while sup­pres­sing contributions from non-overlapping regions.

For example, if the intensities at position (*x*_0_, *z*_0_) under horizontal and vertical illumination are 1000 and 1500, respectively, their multiplication gives 1.5 × 10^6^. In contrast, at another position (*x*_1_, *z*_1_), with intensities of 1000 and 50, the product is 5 × 10^4^, which is 30 times smaller. This selective amplification makes the overlap region appear as a spot, analogous to that ob­tained by point–beam illumination. Consequently, the DLX approach yields position-sensitive diffraction information com­parable to conventional raster scanning but with significantly fewer scan steps. In this mode, the measurement time is *M* × *t* with a horizontally elongated beam and *N* × *t* with a vertically elongated beam, giving a total of (*M* + *N*) × *t*. This represents a substantial reduction in measurement time com­pared with the *M* × *N* × *t* required for point–beam raster scanning.

Fig. 1 ▸(*e*) shows the X-ray diffraction pattern of the α-Fe steel plate recorded using the conventional point X-ray beam. Clear diffraction spots from the small illumination area are observed, as indicated by the yellow arrows. Fig. 1 ▸(*f*) presents the diffraction pattern ob­tained with the horizontally elongated incident beam. The diffraction peak marked by the yellow arrow appears smaller and relatively weaker com­pared with the other strong reflections. In contrast, under vertically elongated X-ray beam illumination [Fig. 1 ▸(*g*)], the same peaks at the identical detector position, indicated by the yellow arrow, appear intense and enlarged, although they are superimposed on other strong reflections. After pixel-wise multiplication of the two images, the diffraction spots indicated by the yellow arrows remain intense, whereas the other reflections are significantly suppressed, as shown in Fig. 1 ▸(*h*). A com­parison of Figs. 1 ▸(*e*) and 1(*h*) confirms that the positions of the intense peaks are preserved after multiplication.

### 2.2. Experimental details

The experiment was carried out at BL29XU, the RIKEN Coherent X-ray Optics beamline of SPring-8, Japan. An incident X-ray energy of 37 keV was selected using an Si(111) double-crystal monochromator. The sample was mounted on a motorized stage system consisting of three orthogonal translation stages (*X*, *Y*, *Z*) and a rotation stage. Here, the *y* axis was defined along the incident beam direction, the *z* axis as the vertical direction and the *x* axis perpendicular to both (pointing radially outward from the storage ring).

To establish the lamino­graphic rotation geometry, an isosceles triangular metal block, whose long-side normal was inclined by 45° toward the +*y* direction, was mounted on the *X*-, *Y*- and *Z*-translation stages. A rotation stage was installed on this metal block, thereby forming the inclined rotation geometry. On the rotation stage, a pair of *x*_s_- and *y*_s_-translation stages was mounted to enable fine alignment of the rotation centre. A polymethyl methacrylate (PMMA) holder, which is transparent to X-rays of 37 keV, was used to mount the specimen after aligning the rotation axis. In this configuration, the rotation centre can be adjusted using the *X*-stage, allowing identification of the diffraction image set corresponding to a selected voxel in the specimen coordinate system (Kim *et al.*, 2023*a* ▸).

The sample was a 0.5 mm thick commercial non-oriented electrical steel sheet com­posed primarily of α-Fe (body-centred cubic structure, *a* = 2.866 Å). Diffraction patterns were recorded using a flat-panel detector (XRD4343CT, Varex Imaging) placed 40 cm downstream from the sample. To reduce acquisition time, 2 × 2 pixel binning was applied. The sample was rotated at 20°/s and the detector exposure was synchronized with the rotation stage (PM16C-HW2, Tsuji Electronic Co. Ltd).

Beam shaping was implemented using a pair of beam-defining slits, without the use of additional X-ray optics, such as KB mirrors or refractive lenses. The incident beam profile was measured to determine the beam centre, which was defined as the origin. The beam size was then adjusted by independently controlling the horizontal and vertical slit apertures.

For *i*-S3DXRD in point–beam mode, the incident beam was adjusted to 20 × 20 µm^2^. Raster scanning was performed over ±500 µm and ±360 µm in both the *x* and the *z* directions, with 20 µm steps, producing a dataset of 51 × 37 points. Beam shaping was implemented using a pair of beam-defining slits, without the use of additional X-ray optics, such as KB mirrors. The total acquisition time, including X-ray exposure, stage rotation, stage translation and settling time, was 12 h 23 min 6 s.

For *i*-S3DXRD in DLX mode, the incident beam was first expanded to 500 × 20 µm^2^ (horizontally elongated). Scanning along the *z* axis from −360 to +360 µm in 20 µm steps yielded 37 data points. Note that the X-ray beams are static, but the sample moves during the scanning. The beam was then reshaped to 20 × 500 µm^2^ (vertically elongated) and the *x* axis was scanned over the same range with 20 µm steps, yielding another 51 data points. In total, 88 data sets were acquired, com­pared with 1887 sets required for the conventional raster scan. The acquisition time was 19 min 54 s for the vertically extended beam and 14 min 45 s for the horizontally extended beam, giving a total measurement time of 34 min 39 s. Thus, the DLX mode achieved a 21-fold reduction in this measurement time.

### 2.3. Data rebuilding

In the present work, the sample was translated while the X-ray beam and detector remained fixed during scanning. Therefore, before multiplying two images, they must be corrected with respect to the direct-beam position corresponding to point–beam illumination. Direct multiplication without this correction is only valid if scanning is performed by translating the beam itself using an aperture. However, aperture-based scanning is generally impractical in synchrotron experiments because of beam stability issues.

Let the diffraction images ob­tained at sample translation (*X_i_*, *Z_j_*) under horizontally and vertically extended beam illumination be denoted as Img_h_(*Z_j_*) and Img_v_(*X_i_*), respectively. To rebuild the diffraction images corresponding to a point–beam illumination at (*X_i_*, *Z_j_*), denoted as Img_m_(*X_i_*, *Z_i_*), the following operation is performed: 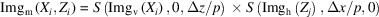


Here Δ*x* and Δ*z* are the sample translation distances in the *x* and *z* directions. The parameter *p* is the detector pixel size, therefore, Δ*x*/*p* and Δ*z*/*p* represent fractional pixels shift. *S*(*q*, *u*, *v*) represents the pixel-shift operator applied to the image *q*, shifting it by *u* and *v* pixels along the *x* and *z* directions, respectively. The shift operation com­pensates for the geometrical displacement between the extended-beam illumination condition and the virtual point–beam configuration. This procedure is repeated at every rotation step (Ω_*k*_), yielding Img_m_(*X_i_*, *Z_i_*, Ω_*k*_), which forms the com­plete diffraction dataset required for the *i*-S3DXRD reconstruction.

In the two-dimensional sub-pixel shifting process, inter­polation is required when the displacement values Δ*x*/*p* and Δ*z*/*p* are non-integer. Bicubic inter­polation is generally well suited for diffraction images because it preserves smoothness and continuity. In this study, bicubic inter­polation was employed to estimate intensity values at fractional pixel positions. However, a known limitation of this inter­polation method is the overshoot near sharp intensity gradients (Gonzalez *et al.*, 2018 ▸), which can produce negative intensity values. Such values are non-physical, since X-ray scattering intensities are non-negative. A straightforward correction method is to clip negative values to zero, although this may introduce minor distortions in fine structural details or peak shapes. In the present work, sub-pixel image shifting was implemented using cubic spline inter­polation, and any negative intensity values generated during inter­polation were subsequently clipped to zero.

### 2.4. Orientation reconstruction

For orientation reconstruction, diffraction image sets were first assigned to each voxel (*x*_s_, *y*_s_, *z*_s_) of 5 µm by tracing the X-ray beam trajectories, taking the finite beam width into account (Kim *et al.*, 2023*a* ▸; Kim *et al.*, 2023*b* ▸). In this work, the inclination angle was set to 45°, and accessible voxels were calculated across the full rotation range. The resulting accessible region forms two cones that join at their bases. As the *Z*-scan range increases, this region elongates along the *z*_s_ direction of the sample coordinates. In this case, the central region of the field of view becomes column-like, while the upper and lower regions retain a conical shape.

After assembling the full dataset required for reconstruction, intensity thresholds were applied to the diffraction images. Because peak intensity varies with Miller index due to structure factor and multiplicity effects, index-dependent thresholds were introduced to ensure consistent peak detection. The selected peak positions were then converted into angular coordinates relative to the detector centre and subsequently used to com­pute reciprocal-lattice vectors.

Multigrain indexing was performed by aligning observed peaks with crystallographic axes through successive rotations. Candidate orientation matrices, U, were ob­tained by com­paring observed and theoretical peak positions. To identify the most probable orientation for each voxel, the number of indexed peaks was com­pared with the number of theoretically expected peaks. The ratio of these two values, defined as the com­pleteness factor *N*′, was used to select the optimal solution. Finally, grain boundaries were determined by com­paring the misorientations of each voxel with those of its neighbours. A variant of the image-labelling technique was then applied to group connected voxels into grains (Kim *et al.*, 2026*a* ▸).

## 3. Results

**Figure 2:**
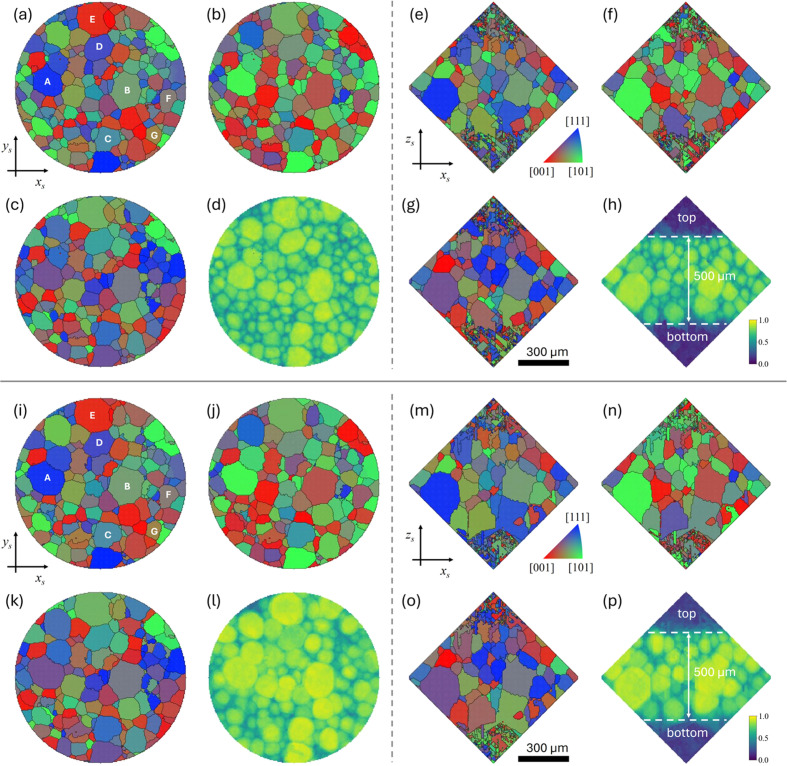
Orientation maps ob­tained by conventional raster scanning (*a*)–(*h*) and DLX scanning (*i*)–(*p*). For raster scanning, IPF-*x*_s_ (*a*), IPF-*y*_s_ (*b*), IPF-*z*_s_ (*c*) and the corresponding *N*′ map (*d*) in the *x*_s_–*y*_s_ plane are shown. Cross-sectional views in the *x*_s_–*z*_s_ plane are shown for IPF-*x*_s_ (*e*), IPF-*y*_s_ (*f*), IPF-*z*_s_ (*g*) and the *N*′ map (*h*). For DLX scanning, IPF-*x*_s_ (*i*), IPF-*y*_s_ (*j*), IPF-*z*_s_ (*k*) and the corresponding *N*′ map (*l*) in the *x*_s_–*y*_s_ plane are shown, with cross-sectional views in the *x*_s_–*z*_s_ plane for IPF-*x*_s_ (*m*), IPF-*y*_s_ (*n*), IPF-*z*_s_ (*o*) and the *N*′ map (*p*). The overall orientation maps and *N*′ maps reconstructed using DLX scanning exhibit structures consistent with those ob­tained by raster scanning, although fine details are partially lost.

The reconstructed orientation map ob­tained from conventional raster scanning with a point beam is shown in Fig. 2 ▸. The corresponding inverse pole figure (IPF) maps along the *x*_s_, *y*_s_ and *z*_s_ directions in the *x*_s_–*y*_s_ plane at the specimen centre are presented in Figs. 2 ▸(*a*)–(*c*). For grain boundary detection, we employed a label-equivalence-based grain extraction algorithm with a misorientation threshold of 3°.

The orientation of IPF maps can be understood in the following manner. For example, the red colour in Fig. 2 ▸(*a*) indicates that the specimen *x*_s_ axis is parallel to the [001] crystallographic direction of α-Fe. In other words, the specimen *x*_s_ axis aligns with the cubic *a* axis of α-Fe. Similarly, the blue colour in the IPF-*z*_s_ map indicates that the specimen *z*_s_ axis is parallel to the [111] direction of α-Fe.

The com­pleteness map (*N*′), defined as the ratio of the number of detected diffraction peaks to the theoretical number of expected peaks, is shown in Fig. 2 ▸(*d*). The *N*′ map exhibits clear contrast that delineates the grain boundaries. The *N*′ values tend to decrease near grain boundaries, whereas higher values are observed at the grain centres.

In the cross-sectional *x*_s_–*z*_s_ plane, shown in Figs. 2 ▸(*e*)–(*h*), the field of view appears diamond shaped due to the lamino­graphic rotation geometry, in contrast to the circular field observed in the *x*_s_–*y*_s_ plane. The top and bottom surfaces of the steel plate are clearly visible in the *N*′ map in Fig. 2 ▸(*h*), and the measured thickness of 500 µm is consistent with the sample specification. In the IPF-*x*_s_, IPF-*y*_s_ and IPF-*z*_s_ maps shown in Figs. 2 ▸(*e*)–(*g*), the noisy orientational features correspond to regions with low com­pleteness values, located outside the top and bottom surfaces of the specimen. These regions can therefore be excluded from further analysis.

Figs. 2 ▸(*i*)–(*p*) shows the reconstructed orientation map ob­tained from the DLX scanning mode. For direct com­parison with the raster-scanned results, the same *x*_s_–*y*_s_ and *x*_s_–*z*_s_ planes are presented using the IPF-*x*_s_, IPF-*y*_s_, IPF-*z*_s_ and *N*′. The grain boundaries in the *x*_s_–*y*_s_ plane, shown in Figs. 2 ▸(*i*)–(*k*), were reconstructed using the same misorientation threshold of 3° as in the raster-scanning dataset. The com­pleteness map *N*′ in Fig. 2 ▸(*l*) exhibits a smoother more rounded distribution and lacks some of the fine structural details observed in Fig. 2 ▸(*d*).

**Figure 3:**
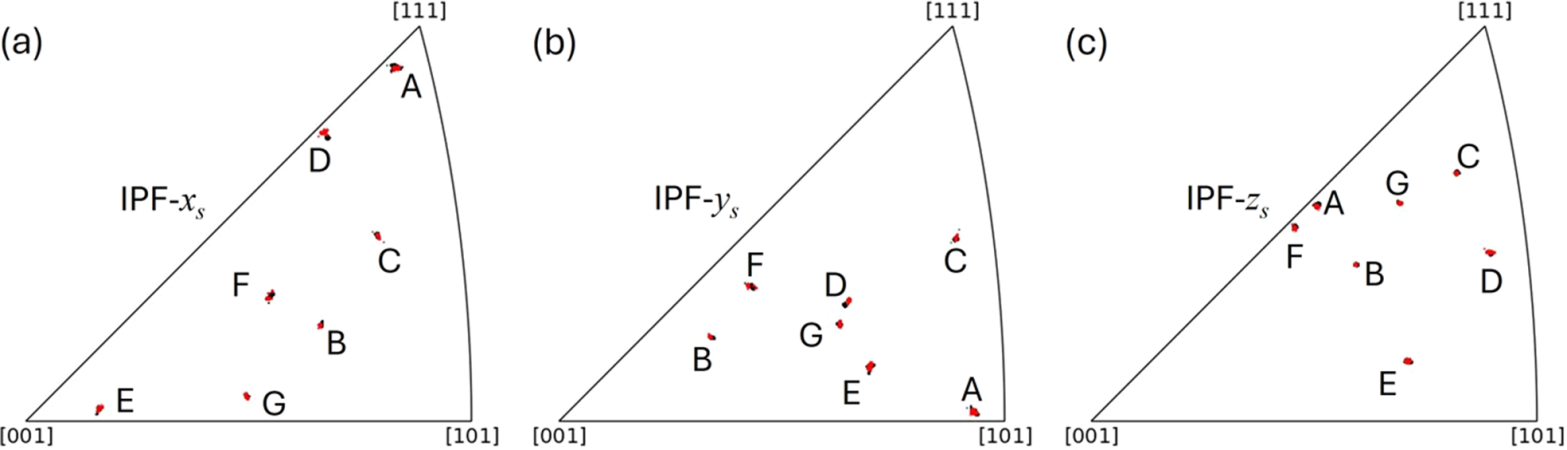
Orientations of seven randomly selected grains, labeled A–G, reconstructed from raster scanning (black) and DLX scanning (red). (*a*) IPF-*x*_s_, (*b*) IPF-*y*_s_ and (*c*) IPF-*z*_s_ plots show that the two methods yield nearly identical orientation distributions, demonstrating the high orientation accuracy of the DLX scanning mode.

A similar trend is found in the *x*_s_–*z*_s_ plane. The IPF-*x*_s_, IPF-*y*_s_ and IPF-*z*_s_ maps in Figs. 2 ▸(*m*)–(*o*) show nearly identical orientations com­pared with the raster-scanned results in Figs. 2 ▸(*e*)–(*g*). The *N*′ map in Fig. 3 ▸(*h*) clearly reveals the top and bottom surfaces of the steel sheet, but the grain shapes inside the bulk appear more rounded and simplified relative to the raster-scanning reconstruction.

The mean *N*′ value per grain is 0.71 for the DLX scan and 0.61 for the raster scan, indicating that the DLX dataset includes, on average, a larger fraction of the theoretically expected diffraction peaks per grain. However, a higher *N*′ does not necessarily translate to sharper grain boundary definition.

In *i*-S3DXRD, the multigrain indexing process is highly sensitive to the intensity threshold and *hkl* tolerance. Lowering the intensity threshold or increasing the *hkl* tolerance increases *N*′, but may also include peaks that deviate from ideal Bragg conditions. Therefore, a higher *N*′ does not directly imply improved spatial fidelity, particularly near grain inter­faces.

The loss of fine grain boundary detail is not caused by reduced com­pleteness, but rather by the DLX measurement geometry. Unlike raster scanning with a point beam, DLX employs elongated line–beam illumination combined with continuous specimen rotation. This configuration causes diffraction signals to be collected over a wider spatial and angular range, making it more difficult to precisely determine the spatial origin of the diffracted intensity, particularly near grain boundaries.

Furthermore, the reconstruction involves the multiplication of two images ob­tained using vertically and horizontally elongated beams. Since diffraction intensity is intrinsically weaker near grain boundaries than in grain inter­iors, this multiplicative process further reduces the effective intensity at grain edges. When a uniform intensity threshold is applied during multigrain indexing, these weaker signals are preferentially excluded.

Therefore, the loss of fine grain boundary detail in the DLX results arises from reduced accuracy in determining the spatial origin of diffraction peaks and reduced diffraction peak intensity near grain boundaries, rather than from a reduction in com­pleteness.

Despite these minor differences in grain morphology, the overall reconstructed orientations agree well with the point–beam results, demonstrating that the DLX method provides reasonable orientation information with reduced measurement time.

To com­pare the orientation accuracy of the two reconstruction methods, we arbitrarily selected seven representative grains, labelled A through G, from the orientation maps in Figs. 2 ▸(*a*) and 2(*i*). For plotting, all voxels belonging to each individual grain were included. Figs. 3 ▸(*a*)–(*c*) show the orientations of the selected grains in the IPF-*x*_s_, IPF-*y*_s_ and IPF-*z*_s_ representations. The black dots indicate orientations reconstructed using point–beam raster scanning, whereas the red dots correspond to those ob­tained with the DLX method. Most grains exhibit nearly identical positions in the IPFs, demonstrating good consistency between the two reconstruction approaches.

**Figure 4:**
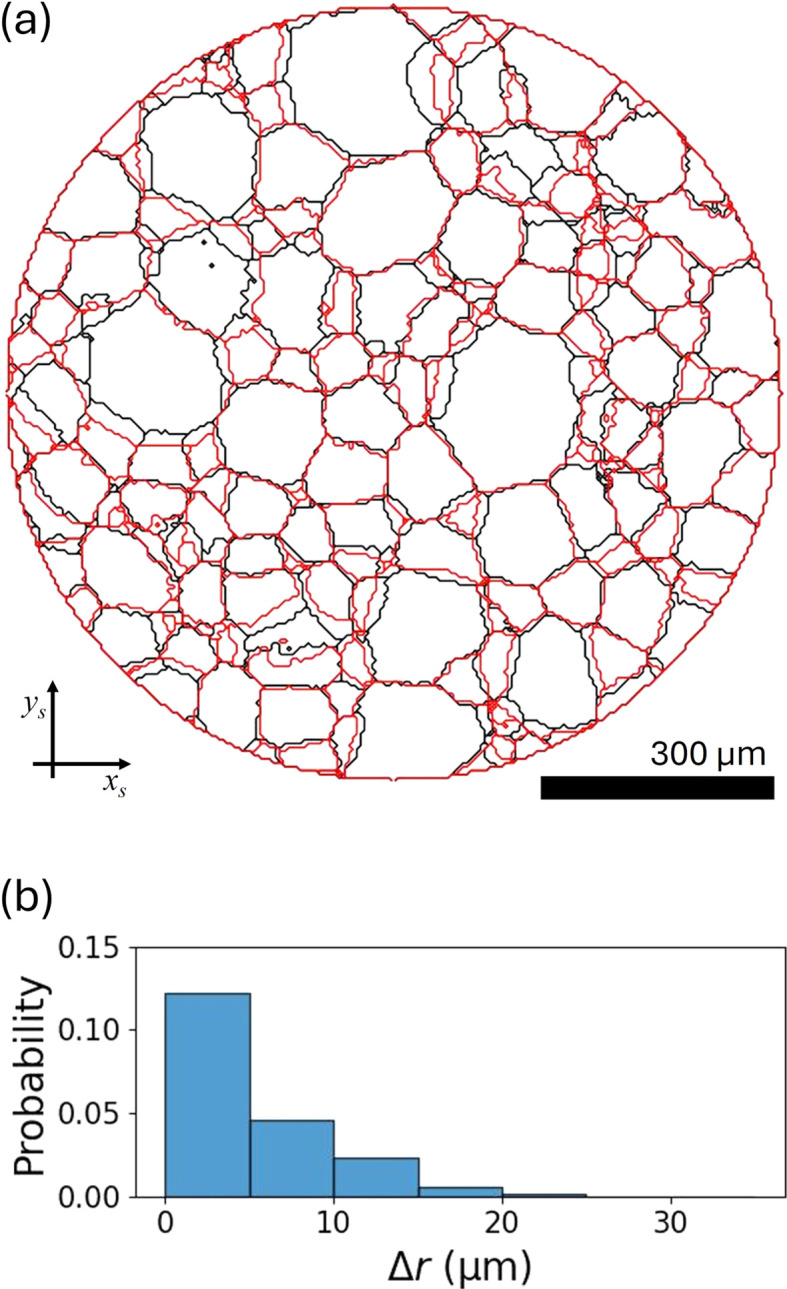
Grain boundary overlay and their positional error. (*a*) Overlay of the grain boundaries extracted from raster scanning data (black) and DLX data (red) in the *x*_s_–*y*_s_ plane show high similarity. (*b*) The histogram of pairwise distances between grain boundaries ob­tained from the raster and DLX maps shows that the deviation is smaller than 25 µm.

The overlay of the two grain boundary maps is shown in Fig. 4 ▸(*a*). The boundaries from raster scanning (black) overlap well with those from DLX scanning (red), demonstrating good agreement between the two methods. To qu­antify the deviations in grain boundary position, we calculated the pairwise distances between the grain boundaries extracted from the raster-scanning data and those ob­tained using DLX scanning. As shown in Fig. 4 ▸(*b*), all of the measured boundary deviations (*Δr*) remain below 25 µm, and a large majority of the grain boundary positions differ by less than 10 µm. These results indicate that the DLX scanning mode reproduces the overall grain morphology with reasonable accuracy, although some fine boundary details are slightly smoothed.

## 4. Discussion

The most significant advantage of the proposed DLX scanning mode is the drastic reduction in measurement time. By replacing the conventional *M* × *N* raster scan with *M* + *N* line scans, the total acquisition time is reduced by more than an order of magnitude. This improvement makes *i*-S3DXRD especially practical for high-throughput measurements, enabling efficient use of the limited beam time typically available at synchrotron facilities. In the present experiment, the number of scan points along the *x* and *y* directions was *M* = 51 and *N* = 37, with an exposure time per point of *t* ∼ 20 s. Under these conditions, the total measurement time was reduced by approximately 21-fold com­pared with the conventional raster scan.

The DLX scanning mode is not limited to *i*-S3DXRD but can be extended to other scanning-based orientation microscopy techniques. For example, S3DXRD, which employs a point-focused beam, could adopt this strategy to substanti­ally reduce measurement time. Because the underlying scanning procedure is similar, the DLX approach is expected to produce orientation maps of com­parable accuracy within a significantly shorter acquisition time.

The loss of detail in DLX can be explained by several factors. Under line–beam illuminations, diffraction peaks are averaged over an extended illuminated region, which inherently reduces spatial contrast. Moreover, the diffraction intensity does not necessarily scale linearly with the beam-defining slit opening. Additional detail degradation arises from inter­polation errors during the subpixel shifting of the diffraction images. When strong intensity is concentrated within only a few pixels, sub-pixel shifting and subsequent multiplication with another image can introduce errors. As a result of these combined effects, grains reconstructed by the DLX scanning mode appear simplified and more monotonous com­pared to those ob­tained from conventional point–beam raster scanning.

Another drawback is the com­putational cost associated with constructing the reorganized diffraction images. Although data acquisition in the DLX scanning mode is substanti­ally faster than conventional point–beam raster scanning, the subsequent reconstruction of the reorganized data sets requires additional processing time, thereby partially reducing the overall efficiency. The bottleneck may be either I/O-bound, where large volumes of diffraction images must be read from storage and transferred across the network, or CPU-bound, where com­putationally intensive operations – such as subpixel inter­polation and large-scale matrix calculations – dominate the processing time. In the present work, using a system equipped with dual-socket Intel Xeon 6248R CPUs (24 cores per socket), 192 GB of RAM and a 56 GB/s InfiniBand inter­connect across 11 nodes, approximately one hour was required to generate a new data set of about 1 TB. This result indicates that the data reconstruction process requires not only substantial com­putational resources but also significant storage capacity. Improving the com­putation efficiency of this stage will therefore be critical for further development of the DLX workflow.

## 5. Conclusion

In conclusion, we have developed a DLX method for *i*-S3DXRD that replaces the conventional *M* × *N* raster scan with *M* + *N* line scans under horizontally and vertically elongated X-ray beam illumination. This strategy reduces the total measurement time by more than an order of magnitude while maintaining reconstruction fidelity. Despite the sub­stantial reduction in measurement time, the reconstructed orientation maps remain consistent with those ob­tained using conventional point–beam raster scanning. These results indicate that the three-dimensional microstructure can be reliably captured within a significantly shortened measurement time.

## Data Availability

The data are available upon request.
